# Effects of Cooling Rate and Solid Fraction on α-Al Phase Evolution in Rheo-Die Casting: Phase-Field Simulation and Experimental Investigation

**DOI:** 10.3390/ma18174169

**Published:** 2025-09-05

**Authors:** Song Chen, Wangwang Kuang, Jian Feng, Hongmiao Wang, Fan Zhang, Daquan Li

**Affiliations:** 1 State Key Laboratory of Nonferrous Structural Materials, China GRINM Group Co., Ltd., Beijing 100088, China; chensong@grinm.com (S.C.); whmhongmiao@163.com (H.W.); 2 GRIMAT Engineering Institute Co., Ltd., Beijing 101407, China; zhangfan@grinm.com; 3 General Research Institute for Nonferrous Metals, Beijing 100088, China; 4 Hongzhiwei Technology (Shanghai) Co., Ltd., Shanghai 200120, China; wwkuang@foxmail.com

**Keywords:** semi solid, rheo-die casting, primary α_1_-Al, secondary α_2_-Al, phase field model, cooling rate, microstructure, solid fraction

## Abstract

This study aims to bridge the critical knowledge gap in understanding the dynamic microstructural evolution during high-solid-fraction semi-solid rheo-die casting process, including slurry preparation (0.1–0.3 K/s) and rheo-die casting (10–150 K/s). A novel phase-field model coupling continuous cooling with explicit nucleation was developed, enabling the dynamic simulation of continuous solidification microstructure evolution, considering two-stage cooling rate transition characteristics. Integrated the Swirled Enthalpy Equilibration Device (SEED) slurry preparation and graded-cooling mold experiments established variable cooling rate and solid fraction conditions for quantitative analysis of α-Al morphological evolution during rheo-die casting solidification. Through experimental and simulation investigations of the Al-7Si alloy, it is concluded that during Stage I slurry preparation, the primary α_1_-Al phase coarsened due to Ostwald ripening. In Stage II rheo-die casting, primary α_1_-Al undergoes continued growth under a moderate cooling rate (15 K/s). Meanwhile, secondary α_2_-Al formation exhibits a cooling-rate and solid fraction dependence: a high cooling rate (150 K/s) promotes explosive nucleation with the volume fraction decreasing from 4.78% to 0.33% as the solid fraction rises, whereas a mid-cooling rate (15 K/s) substantially suppresses its formation. Mechanistically, a high cooling rate promotes solute trapping, which intensifies constitutional undercooling, thereby elevating both the nucleation and growth driving forces to facilitate the formation of secondary α_2_-Al, whereas higher solid fractions restrict secondary phase formation by narrowing the solidification windows from 22 °C to 7 °C.

## 1. Introduction

Compared to traditional liquid metal forming, high-solid-fraction semi-solid rheo-die casting of aluminum alloy represents a transformative advancement, achieving synergistic optimization of near-net-shaping and high performance [1,2]. This process promotes non-dendritic microstructure formation and laminar filling behavior of high-viscosity slurry to suppress gas entrapment and oxide inclusion, which can be strengthened with solution treatment [3,4,5]. Consequently, this innovative technique has recently achieved widespread adoption in the automotive industry [6,7].

The solidification process of high-solid-fraction semi-solid rheo-die casting exhibits a unique dual-stage cooling characteristic [8,9]. Firstly, slurry preparation (Stage I) at near-liquidus temperature (600–610 °C) with a cooling rate of 0.1–0.3 K/s forms high-solid-fraction slurries (30–50%) containing near-spheroidal primary α_1_-Al. Secondly, the slurries undergo a cooling-rate transition (a two-order magnitude increase) in the rheo-die casting process (Stage II). While primary α_1_-Al nucleation and growth have been extensively investigated [10,11], the dynamic microstructure evolution during high-solid-fraction slurry preparation remains inadequately researched. Crucially, microstructure characterization consistently confirms the presence of secondary α_2_-Al phase in the semi-solid metal (SSM) process. Ji et al. [12] found the regulatory mechanism of shear fields on residual liquid solidification in the low-melting-point Sn-Pb model alloy. Under high shear rates (>5200 s^−1^), a spherical secondary phase (size ≈ 5 μm) forms in the residual liquid, overturning conventional dendritic growth paradigms. This foundational work established the theoretical basis for secondary α_2_-Al studies. Hitchcock et al. [13] studied the influence of intensive shearing on the solidification behavior of the remaining liquid and its effect on tailoring the final microstructure. They found that during the secondary solidification process within the die cavity, the α-Al phases (α_3_) grew spherically from independent nuclei rather than forming dendritic fragments or interconnected dendritic structures. Concurrently, Liu et al. [14] established quantitative correlations between pouring temperature/cooling rate and secondary α_2_-Al grains, using the Vibrated Contraction Slope Plate (VCSP) technology. However, these static characterization approaches cannot resolve in situ microstructure evolution at specific solid fractions, nor quantify the coupling effects of cooling-rate transitions and solid fraction variations. Moreover, although the persistent presence of the secondary α_2_-Al phase has been consistently documented in semi-solid metal (SSM) processing, prior investigations have not systematically addressed the coupled effects of cooling-rate transitions and solid-fraction variations on its formation kinetics and dynamic evolution.

Theoretical simulation of microstructure and solute evolution during semi-solid casting provides critical insights into solidification mechanisms. Phase-field modeling, which utilizes order parameter fields to describe phase distribution without explicit interface tracking, offers unique advantages for simulating complex morphological evolution and has been widely adopted for solidification studies [15,16,17]. Recent advances in multiscale phase-field simulations for semi-solid metal (SSM) have enabled unprecedented visualization of microstructure dynamics. Qu et al. [18,19] developed a phase-field lattice Boltzmann coupled model for semi-solid slurries, revealing grain evolution mechanisms under complex boundary conditions, particularly the transformative effects of rotational external fields. Guo et al. [20] modified a KKS multi-field model (integrating phase-field, solute diffusion, and thermal transport) to quantify competitive growth kinetics in dendritic structures. Li et al. [21] investigated coarsening behavior under varying grain size distributions using phase-field methods. Das et al. [22] studied microstructure evolution in cooling-slope-processed semi-solid slurries. Qin et al. [23] employed the phase-field method integrated with the thermodynamic database MTDATA to simulate the growth process of Al_3_Fe_2_ particles from an Al-Fe-Si melt under isothermal conditions in semi-solid processing.

However, the above studies on phase-field modeling of the SSM process face two critical limitations: Firstly, conventional simulations operate at restricted spatiotemporal scales. Conventional SSM phase-field simulations commonly rely on the input of thermodynamic and kinetic parameters corresponding to a fixed composition and a near-isothermal state to simulate the evolution of microstructure. And the simulation scale of the microstructure is significantly different from that of the actual volume. High-solid-fraction semi-solid rheo-die casting involves complex microstructure evolution processes that typically last from tens to hundreds of seconds. Moreover, this technique needs to account for a wide range of grain sizes, spanning from several micrometers to hundreds of micrometers. Secondly, conventional phase-field models for semisolid processing fail to capture the spontaneous nucleation behavior of α_2_-Al phases—especially under the high cooling rates (1–200 K/s) characteristic of Stage II in rheo-die casting—which are nearly two orders of magnitude higher than those during slurry preparation (0.1–0.3 K/s). These models particularly lack descriptions for spontaneous nucleation and growth kinetics of secondary α_2_-Al under the high cooling rate of Stage II.

In this study, we aim to bridge the critical knowledge gap in understanding the dynamic microstructure evolution during high-solid-fraction semi-solid rheo-die casting. We have developed a phase-field model for high-solid-fraction semi-solid rheo-die casting by extending the classical Karma-Rappel framework [24]. The enhanced model incorporates continuous cooling algorithms with explicit nucleation methods to accurately capture spontaneous nucleation during solidification, while specifically accounting for cooling rate transitions and high-solid-fraction conditions. Using an Al-7Si alloy as a model system, our integrated experimental and computational approach systematically investigates the evolution mechanisms of α_1_-Al phases under varying processing conditions, with particular emphasis on understanding the nucleation dynamics and growth kinetics of secondary α_2_-Al during rheo-die casting. Meanwhile, Al-7Si is one of the most commercially significant semi-solid casting alloys. This mechanistic insight provides a theoretical foundation for precisely tailoring microstructure in semi-solid processing. Furthermore, this study method and novel phase field model are expected to be extended to other Al-Si and Al-based alloy systems designed for semi-solid processing.

## 2. Materials and Methods

### 2.1. Experiment Methods

Al-7Si binary alloy was chosen as the target material to reveal the microstructure evolution of α-Al due to exceptional cast fluidity and comprehensive mechanical properties. The alloy was prepared by melting commercially pure Al (99.7 wt.%) and Al-20Si master alloy in a resistance furnace (FOSECO, Beijing, China). After degassing with a rare-earth agent at 720–740 °C, the high-purity melt was cast into a special steel mold for composition analysis. The actual chemical compositions were determined by an inductively coupled plasma atomic emission spectrum (ICP-AES) apparatus (SPECTRO ARCOS, SPECTRO, Kleve, Germany), and the results are shown in Table 1.

**Figure 1:**
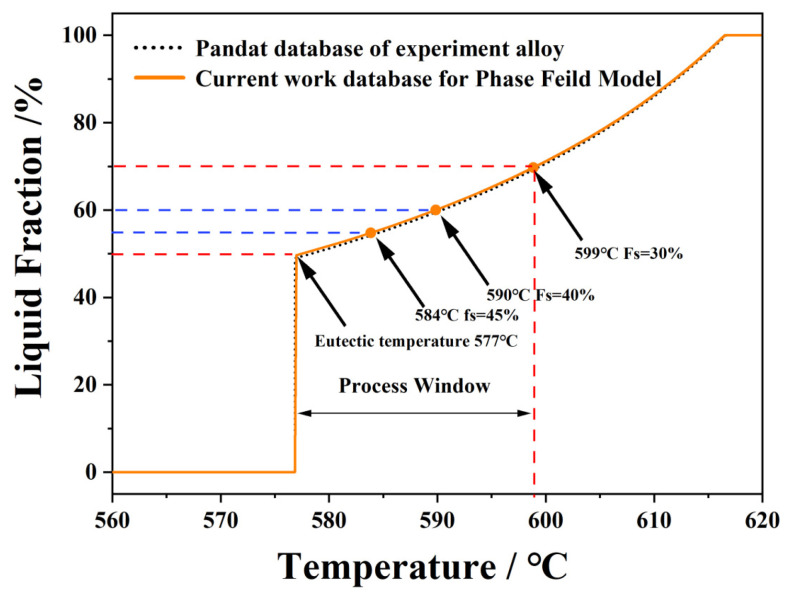
Solidification path of Al-7Si alloy: evolution of solid fraction (Fs) vs. temperature (T).

Solid fraction-temperature profile calculated via Calculation of Phase Diagrams (CALPHAD) methodology [25,26] is presented in Figure 1. The theoretical solid fraction reaches 48% at the eutectic temperature of 577 °C. Considering the exothermic effects during eutectic transformation that compromise slurry stability, three pre-eutectic solid fractions (30%, 40%, and 45%) corresponding to temperatures of 598 °C, 589 °C, and 580 °C were selected for high-solid-fraction slurry preparation.

**Figure 2:**
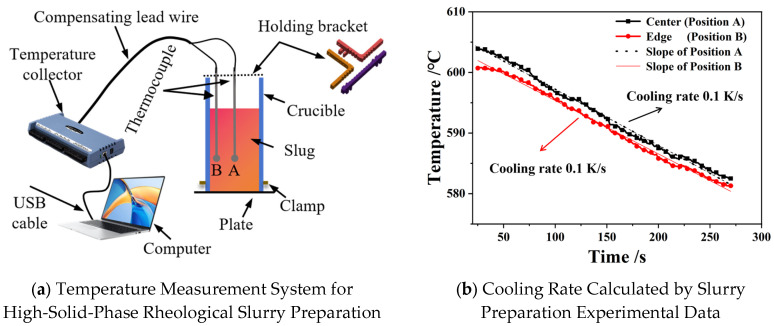
Temperature monitoring system and experimental cooling curves for high-solid-fraction slurry preparation (Stage I). (**a**) Temperature monitoring system for high-solid-fraction rheological slurry preparation; (**b**) Cooling rate experimental data calculated by slurry preparation (0.10 K/s).

**Figure 3:**
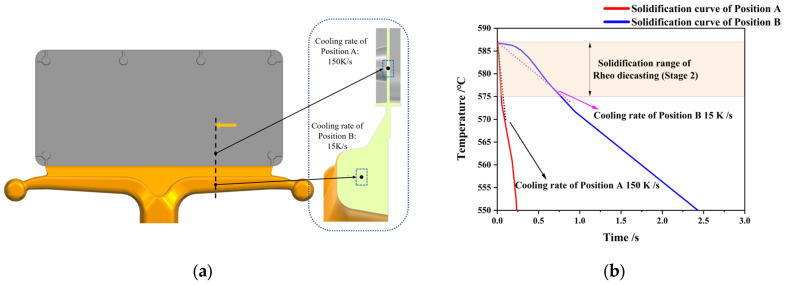
Sampling points in 3D gating system and corresponding ProCAST-simulated solidification cooling curves during semi-solid rheo-die casting filling for various mold locations. (**a**) Gating system and sampling point of experiment mold for rheo-die casting; (**b**) Temperature distribution contour and derived by fitting cooling rates of ProCAST simulated.

During the primary cooling stage (Stage Ⅰ), high-solid-fraction semi-solid slurries were prepared using the Swirled Enthalpy Equilibration Device (SEED) method. Solid fraction development was determined through correlation with the equilibrium diagram (Figure 1). Molten alloy was poured at 635 °C into an open crucible (Figure 2a). Rheo-slurries with varied solid fractions were obtained by controlling the rotation time. Temperature evolution was monitored in situ via thermocouples, and the solidification curves are shown in Figure 2b. Crucially, the cooling rate during slurry preparation is defined as the primary cooling rate, while the solidification cooling rate during the rheo-die casting process (Stage II) is defined as the secondary cooling rate. Condition-controlled slurries were subsequently subjected to rheo-die casting involving distinct cooling-rate transitions. To elucidate the role of different secondary cooling rates in α-Al phase evolution, two typical sampling locations were designated at a runner (10 mm wall thickness) and a thin-wall plate (0.7–0.8 mm wall thickness). These samples were from the same locations as the optical microscope/scanning electron microscope (OM/SEM) samples shown in Figure 3a. The OM/SEM microstructure samples were cut using a vertical band sawing machine and mounted with dental tray powder. Subsequently, the samples were ground with SiC sandpaper of 400#, 1000#, and 2000# grit, followed by standard mechanical polishing and etching in Keller’s reagent (2 mL HF, 3 mL HCl, 5 mL HNO_3_, 190 mL H_2_O). Microstructural observation was carried out using an Axiovert 200 MAT microscope (Carl Zeiss, Oberkochen, Germany). Three micrographs (100× and 200× magnification) were randomly acquired from each sample for quantitative analysis. The maximum phase size (dmax) and area fractions of α-Al phases were measured using Image-Pro Plus 6.0 software (Media Cybernetics, Inc., Rockville, MD, USA). SEM observations and Energy Dispersive X-ray Spectroscopy (EDS) analysis were performed using a ZEISS Sigma 300 brand (Carl Zeiss, Oberkochen, Germany) equipped with an Oxford Ultim Max65 energy-dispersive X-ray spectrometer (EDS; Oxford Instruments, Oxford, UK). The SEM was operated at an accelerating voltage of 20 kV, magnifications of 500× and 1000×, and a working distance of 10–15 mm. Secondary cooling rates were determined through ProCAST simulations (ESI Group, Paris, France, 2009), where temperature evolution during filling and solidification was modeled. Temperature-time profiles were extracted at sampling points and fitted to derive cooling rates. Figure 3b presents region-specific simulated cooling curves with corresponding fitted cooling rates.

In summary, the primary cooling rate of Stage I was determined through experimental thermometry under actual process conditions, while the secondary cooling rate of Stage II was extracted from finite-element simulations of temperature evolution. These cooling rates served as critical input parameters for phase-field modeling, enabling a multiscale framework that bridges macro-scale temperature fields with mesoscale nucleation and microstructure evolution. This approach significantly enhances the physical fidelity of phase-field simulations to actual solidification processes, establishing a foundation for accurately revealing microstructure evolution in high-solid-fraction semi-solid rheo-die casting.

### 2.2. Phase Field Model

In the phase field model, there are two types of field variables: non-conserved fields and conserved fields. In solidification problems, the non-conserved field is used to describe the distribution of phases, which can be simply regarded as the phase volume fraction, and its evolution is described by the Allen-Cahn equation [27]; the conserved field is generally the solute concentration, described by the Cahn-Hilliard equation [16].

The solidification of alloys involves both heat transfer and mass transfer processes. During isothermal solidification, the evolution of the interface largely depends on the solute concentration fields on both sides of the liquid-solid interface. The distribution and evolution of the solute field are governed by the classical mass conservation equation (Fick’s law [28]). To simulate the growth process of the solid phase, an order parameter ϕ is defined in space, representing the local solid phase fraction. ϕ=1 represents a pure solid phase. ϕ=0 indicates liquid phase. Within the solid-liquid interface layer, its order parameter ϕ varies continuously between 0 and 1. For the solid phase, ϕ, the control equation of its variational phase field mode (ϕ) can be expressed as [29,30]:

(1) $$\tau \left[1+\left(1-k\right)u\right]a_{s}^{2}\left(\vec{n}\right)\partial_{t}\phi =W^{2}\nabla \cdot \left[a_{s}^{2}\left(\vec{n}\right)\nabla \phi \right]+W^{2}\partial_{x}\left[{\left|\nabla \phi \right|}^{2}a_{s}\left(\vec{n}\right)\frac{\partial a_{s}\left(\vec{n}\right)}{\partial \left(\partial_{x}\phi \right)}\right]+W^{2}\partial_{y}\left[{\left|\nabla \phi \right|}^{2}a_{s}\left(\vec{n}\right)\frac{\partial a_{s}\left(\vec{n}\right)}{\partial \left(\partial_{y}\phi \right)}\right]+W^{2}\partial_{z}\left[{\left|\nabla \phi \right|}^{2}a_{s}\left(\vec{n}\right)\frac{\partial a_{s}\left(\vec{n}\right)}{\partial \left(\partial_{z}\phi \right)}\right]-2\phi \left(1-\phi \right)\left(1-2\phi \right)-8\lambda u\phi^{2}{\left(1-\phi \right)}^{2}$$

For the solute concentration c (mole fraction), its governing equation is [18]:

(2) $$\partial_{t}c=\nabla \cdot \left\{D_{\phi }\left[\nabla c+c\frac{1-k}{1-\left(1-k\right)\phi }\nabla \phi \right]-\frac{Wc}{\sqrt{8}}\frac{\left(1-k\right)}{1-\left(1-k\right)\phi }\partial_{t}\phi \frac{\nabla \phi }{\left|\nabla \phi \right|}\right\}$$

where

(3) $$D_{\phi }=D_{l}\left(1-\phi \right)+D_{s}\phi$$

is the effective diffusion coefficient, $D_{l}$ is the liquid-phase diffusion coefficient, $D_{s}$ is the solid-phase diffusion coefficient, and k is the equilibrium solute partition coefficient of the solid phase.

(4) $$u=\frac{\frac{c-kc_{l}^{eq}}{1-\left(1-k\right)\phi }+1}{1-k}$$

is the solute supersaturation for the solid phase, where $c_{1}^{eq}$ is the equilibrium liquid-phase concentration, and both k(*T*) and $c_{1}^{eq}(T)$ are obtained from the phase diagram calculation in the current work.

$a_{s}\left(\vec{n}\right)$ is the anisotropy function. In this paper, two-dimensional simulation is carried out, and the following form is adopted: $a_{s}\left(\vec{n}\right)=1+\varepsilon cos\left[m\left(\theta -\theta_{0}\right)\right]$, where ε is the anisotropy strength, m is the symmetry degree, and 4 for *fcc* symmetry is adopted in the present work due to the *fcc* structure of α-Al.

$W=\lambda d/a_{1}$ is interface characteristic length of the solid phase where *d* is the solute diffusion capillary length of the solid phase and λ is the coupling constant of the solid phase.

τ is the time relaxation coefficient of the solid phase. According to the KR model, it can be expressed as

(5) $$\tau =a_{2}\lambda W^{2}/D_{1}$$

$a_{1}=5\sqrt{2}/8$, $a_{2}=47/75$, which are constants [18]. Note that the first term on the right side of Equation (2) is the diffusion term, and the second term is the anti-trapping current term.

In this study, we introduce the solid phase nucleation mechanism based on an explicit nucleation algorithm. This method essentially involves randomly generating nucleation sites during the simulation, which has a strong artificial control characteristic, but the nucleation rate is described by classical nucleation theory, making it physically meaningful. The explicit nucleation algorithm was first proposed by Simmons et al. [31]. It allows for the random generation of nucleation sites in the simulation, but the undercooling at these sites must exceed a critical undercooling for grain nucleation to occur. As the temperature further decreases, the initially nucleated grains will grow with increasing undercooling, accompanied by the nucleation of new grains in other regions.

For a single nucleation site, according to classical nucleation theory, its nucleation rate should be satisfied by the equation [32]:

(6) $$J=Z\beta^{*}exp\left(\frac{-\Delta G^{*}}{k_{B}T}\right)exp\left(\frac{-\tau_{N}}{t}\right)$$

Therein, *Z* is the Zeldovich factor, $\beta^{*}$ is the frequency factor for the growth of the critical nucleus into a supercritical nucleus, $\Delta G^{*}$ is the activation energy of the critical nucleus related to local composition and temperature, $\tau_{N}$ is the nucleation incubation time, $k_{B}$ is the Boltzmann constant, and T is the temperature.

It can be observed that the above equation has a complex form, containing many difficult-to-estimate parameters, which makes it inconvenient for use in phase-field simulations. Therefore, based on its structural characteristics, it is simplified as follows:

(7) $$\begin{array}{c} J=k_{1}exp\left(\frac{-k_{2}}{u}\right) \end{array}$$

*k*_1_, *k*_2_ are constants. Equation (7) retains the basic meaning of Equation (6), but simplifies multiple parameters into two adjustable parameters, achieving consistency with experimental results by regulating *k*_1_ and *k*_2_. Furthermore, the local nucleation rate of the phase position is calculated using the following formula:

(8) $$\begin{array}{c} P=1-exp\left(-J\Delta t\right) \end{array}$$

During the simulation process, at potential nucleation sites, a random number R is generated between $\left[0,1\right]$. Once R<P is found, a nucleation point is introduced at that location, the order parameter ϕ at that position is set to 1.

To accurately describe the continuous cooling process, our phase-field model synchronously couples with the CALPHAD (Calculation of Phase Diagrams) method [25]. This integration enables real-time acquisition of temperature-dependent parameters during cooling, including the equilibrium solute partition coefficient k and the equilibrium liquid concentration, with all simulation parameters retrieved from the thermodynamic databases. For the Al-Si system, the thermodynamic database referenced in [26] was employed. Based on this database, the Al-Si phase diagram presented in Figure 1 shows that α-phase solidification initiates at 616.77 °C and progresses to the eutectic solidification temperature of 576.92 °C. Due to the unavailability of temperature-dependent diffusion coefficients, these parameters are treated as constants. All simulation parameters are listed in Table 2.

**Figure 4:**
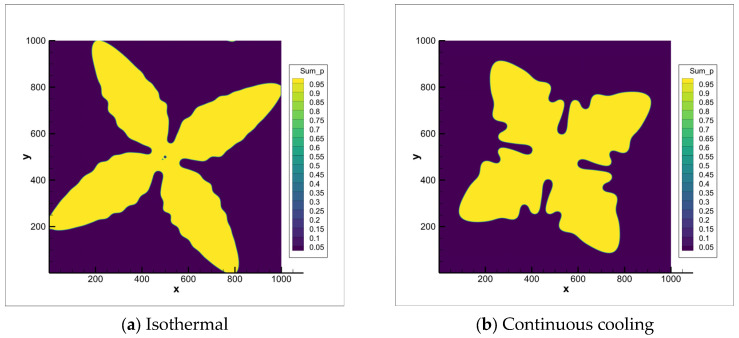
Phase-field simulation results comparing primary phase growth to 35% solid fraction under single nucleation site conditions: (**a**) Isothermal solidification; (**b**) Continuous cooling process.

Given the substantial capillary length adopted in this model, the interfacial characteristic length W, as defined by its governing equation, occurs on the micrometer scale. Within the phase-field framework utilizing these parameters, the influence of cooling conditions on solidification dendrite morphology was first investigated. Pronounced dendritic features are observed in grains formed under isothermal solidification, as revealed in Figure 4a. In contrast, a microstructure exhibiting significantly increased equiaxity is observed during continuous cooling (Figure 4b). This difference fundamentally arises from distinct transformation kinetics: continuous cooling initiates phase transformation at higher temperatures, where the weaker initial thermodynamic driving force permits more extensive solute redistribution prior to significant interface advancement. Conversely, isothermal growth commences directly at a lower temperature, imposing a substantially higher driving force that consequently amplifies anisotropic growth tendencies. The model’s inherent capability to accurately simulate continuous cooling scenarios renders it directly applicable for modeling practical semi-solid forming processes, as will be detailed in the subsequent chapter.

## 3. Results and Discussion

### 3.1. Rheo-Die Casting Experiment Results

**Figure 5:**
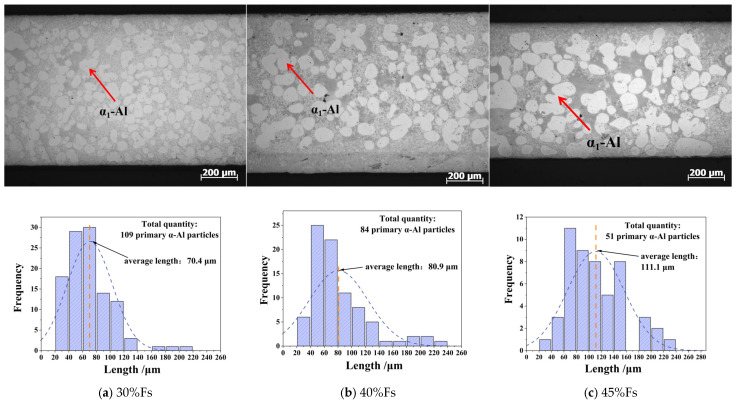
OM microstructure and statistics results of primary α_1_-Al phase with differential solid fractions for Al-7Si alloy: (**a**) 30% Fs, (**b**) 40% Fs, (**c**) 45% Fs.

To accurately characterize the primary α_1_-Al phase with different solid fractions, optical microscope (OM) samples were extracted from regions experiencing a high cooling rate (150 K/s), as specified in Figure 3a. Figure 5 shows the OM microstructure of different solid fractions at 150 K/s. It is obvious that the matrix mainly consists of the primary α_1_-Al phase, secondary α_2_-Al phase with fine and dispersed white dots, and eutectic Si phase with a gray zone. When the solid fraction increases from 30% to 45%, the average length of the α_1_-Al phase increases from 70.4 μm to 111.1 μm. In other words, discrete spheroidal primary α_1_-Al phases exhibit coalescence tendencies toward larger grains through Ostwald ripening.

**Figure 6:**
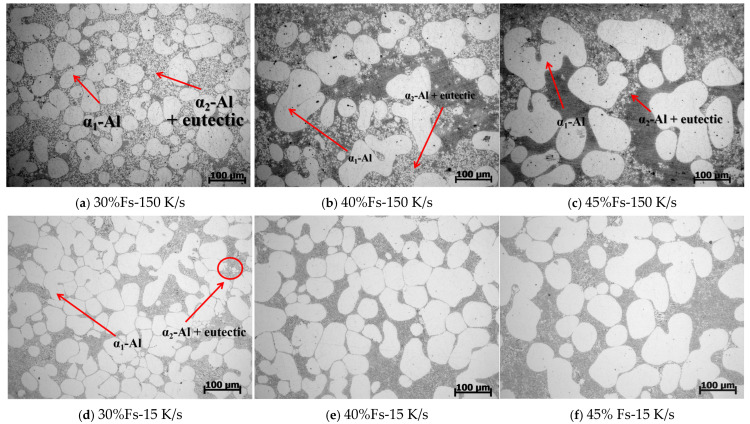
Higher magnification OM microstructures of semi-solid rheo-die casting Al-7Si alloy with differential solid fractions and cooling rates: (**a**) 30% Fs-150 K/s, (**b**) 40% Fs-150 K/s, (**c**) 45% Fs-150 K/s, (**d**) 30% Fs-15 K/s, (**e**) 40% Fs-15 K/s, (**f**) 45% Fs-15 K/s.

Figure 6 shows the high-magnification microstructure of semi-solid rheo-die cast specimens under different cooling rates. At the high cooling rate (150 K/s), some primary α_1_-Al phases change from near-spherical to rosette morphology as solid fractions increase (Figure 6a–c). Many secondary α_2_-Al phases appear around the primary α_1_-Al phases. Both the number and size of the secondary α_2_-Al phases decrease as the solid fraction increases. These phases also tend to accumulate near primary α_1_-Al interfaces. At the mid-cooling rate (15 K/s), the primary α_1_-Al grains grow larger with increased solid fractions (Figure 6d–f). This matches the trend in Figure 5. At the low solid fraction (30%), very few secondary α_2_-Al phases form between primary grains (Figure 6d). These phases appear larger but are fewer in number than in high-cooled samples (Figure 6a,d). At higher solid fractions (40–45%), almost no secondary α_2_-Al phases appear near primary grains (Figure 6e–f). Importantly, at solid fractions of 40% and 45%, the higher-cooled specimens (150 K/s) still contain secondary α_2_-Al. Mid-cooled (15 K/s) specimens show almost no α_2_-Al phases at these conditions.

**Figure 7:**
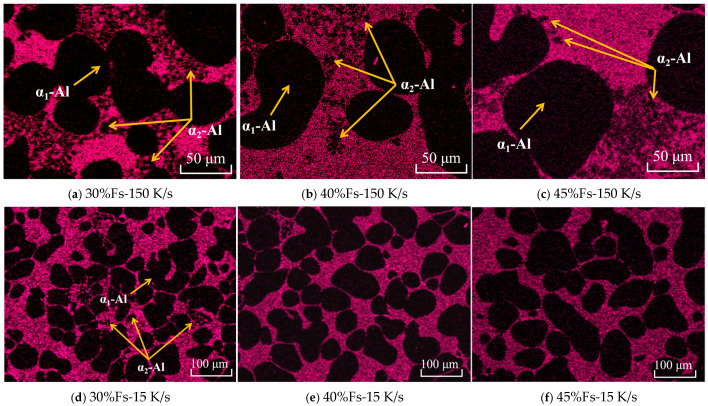
The Si element of SEM mapping of semi-solid rheo-die casting Al-7Si alloy with different solid fractions and cooling rates: (**a**) 30% Fs-150 K/s, (**b**) 40% Fs-150 K/s, (**c**) 45% Fs-150 K/s, (**d**) 30% Fs-15 K/s, (**e**) 40% Fs-15 K/s, (**f**) 45% Fs-15 K/s.

To confirm the presence of small phases in the residual eutectic regions, the Si element of SEM/EDS mapping was performed on the samples with different solid fractions and cooling rates, and the results are shown in Figure 7. The α-Al phase is shown in black, and the eutectic Si phase is shown in pink. At the higher cooling rate of 150 K/s with a 30% solid fraction (Figure 7a), many fine secondary α_2_-Al phases are uniformly dispersed around primary α_1_-Al grains within the eutectic regions. Both the number and size of these phases decrease as the solid fraction increases. Under the mid-cooling rate of 15 K/s, secondary α_2_-Al phases appear in eutectic regions only at low solid fractions (Figure 7d). Primary α_1_-Al grains show significant growth and coalescence with increased solid fractions (Figure 7e,f). These results are consistent with the observations in Figure 5 and Figure 6.

### 3.2. Phase Field Simulation Results

**Figure 8:**
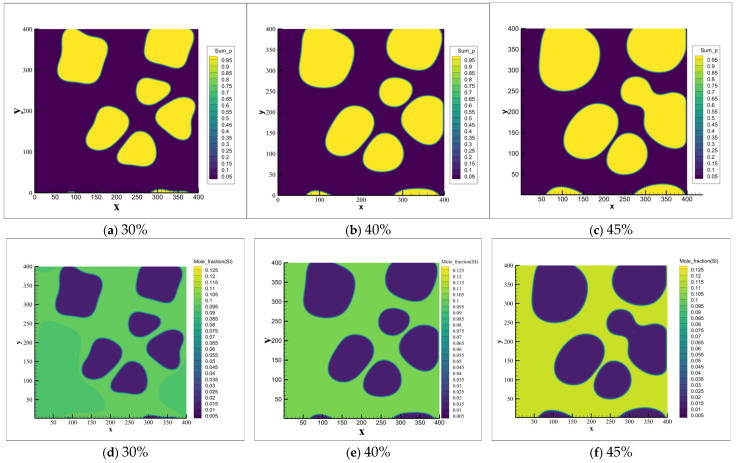
Phase/solute field evolution of primary α-Al during semi-solid slurry preparation (0.1 K/s) at 30%, 40%, and 45% solid fractions. (**a**–**c**) Phase field: (**a**) 30%, (**b**) 40%, (**c**) 45%; (**d**–**f**) Solute field: (**d**) 30%, (**e**) 40%, (**f**) 45%.

Based on the experimental observations, the primary α_1_-Al grains formed during slurry preparation reach sizes of several tens of micrometers. To facilitate direct comparison with the experimental results, the phase-field simulation domain was set to 400 μm × 400 μm with a 1 μm grid resolution. Since no secondary α_2_-Al formation was observed during slurry preparation cooling experiments [8,10,11], the stochastic nucleation module was disabled in the Stage I simulations. Instead, six nucleation sites were randomly initialized within the computational domain. The simulated evolution of the primary α_1_-Al phase during slurry preparation (Stage I) is presented in Figure 8. As the solid fraction increases, grains progressively grow and exhibit coalescence tendencies, which is consistent with the experimental morphology shown in Figure 5. It is confirmed that the liquid concentration gradually increases with increasing solid fraction due to sufficient diffusion time under a low cooling rate (0.1 K/s).

**Figure 9:**
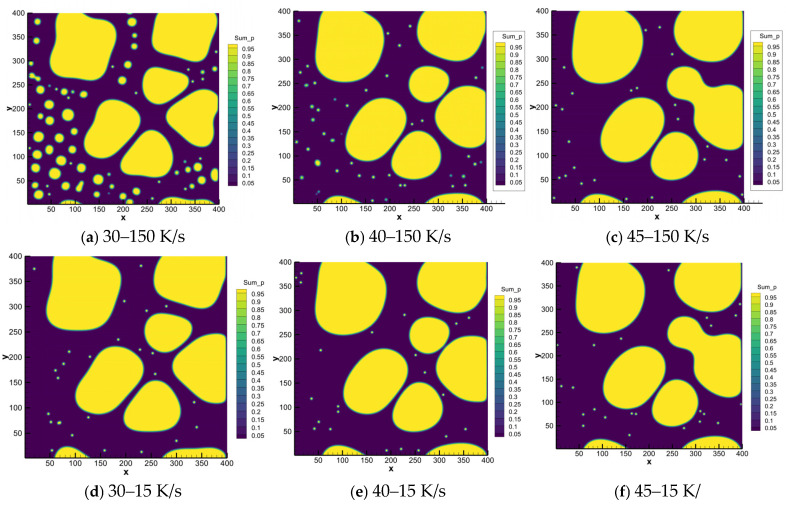
Phase-field simulation of secondary α-Al nucleation/growth behavior with different solid fractions and cooling rates: (**a**) 30% Fs-150 K/s, (**b**) 40% Fs-150 K/s, (**c**) 45% Fs-150 K/s, (**d**) 30% Fs-15 K/s, (**e**) 40% Fs-15 K/s, (**f**) 45% Fs-15 K/s.

Subsequently, we initialized phase-field simulations for Stage II of rheo-die casting, using the final slurry-preparation state. The spontaneous nucleation module was activated to study the microstructure evolution during this stage. This approach focuses on revealing the formation mechanisms of the α_2_-Al phase. Figure 9 presents the simulation results for fixed Stage II cooling rates (150 and 15 K/s) with varied solid fractions. Higher cooling conditions (150 K/s) significantly promote secondary α_2_-Al formation. These spherical secondary α_2_-Al phases disperse in the residual liquid near the primary grains. Increasing the solid fraction from 30% to 45%, the volume fraction decreases from 4.78% to 0.33% and the nucleation density simultaneously decreases from 62 to 20 nuclei. Moreover, the phase size also reduces at higher solid fractions. Comparing with the mid-cooling rate of 15 K/s, the secondary α_2_-Al formation remains nearly absent at 15 K/s but increases substantially at 150 K/s. These simulation results align with the experimental observations in Figure 6.

### 3.3. Microstructural Evolution Mechanisms of Primary and Secondary α Phase During Semi-Solid Rheo-Die Casting Process

**Figure 10:**
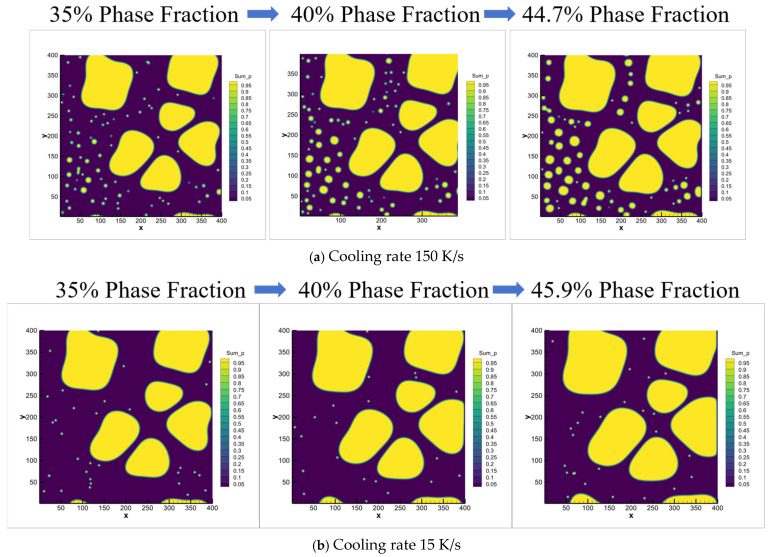
Phase-field simulated dynamic evolution of secondary α_2_-Al phase during rheo-die casting from 30% solid-fraction slurry under differential cooling rates. (**a**) 150 K/s, (**b**)15 K/s.

Based on the experimental and simulated microstructural evolution, the primary α_1_-Al phase predominantly grows in spherical morphology during Stage II rheo-die casting. During the semi-solid slurry preparation (Stage I), as the solid fraction increases in the Al-7Si alloy, adjacent primary α_1_-Al grains undergo Ostwald ripening [22], progressively coalescing into rosette configurations to minimize system energy in the Al-7Si alloy. Figure 10 further illustrates the dynamic evolution of the microstructure at a fixed Stage I solid fraction of 30% under varying cooling rates. The formation of secondary α_2_-Al is nearly absent at the medium cooling rate of 15 K/s, but dramatically increases at the high cooling rate of 150 K/s, consistent with the experimental data in Figure 6.

Regarding nucleation/growth mechanism of secondary α_2_-Al following a cooling-rate transition, this study focuses on the process-dependent dynamics. Nucleation follows an explosive pattern driven by non-equilibrium conditions, as governed by classical theory (Equation (6)).

Therein, the critical nucleation energy barrier $\Delta G^{*}$(J/m^3^) is given by Equation (9),

(9) $$\Delta G^{*}=\frac{16\pi \gamma^{3}}{3(\Delta G_{v}{)}^{2}}$$

where γ is the solid-liquid interfacial energy (J/m^2^) and $\Delta G_{v}$ is the volumetric free energy difference between the liquid and solid:

(10) $$\Delta G_{v}\approx \frac{L_{v}\Delta T_{eff}}{T_{m}}$$

where $L_{v}$ is the solidification enthalpy of per unit volume (J/m^3^) and $T_{m}$ is the equilibrium melting temperature (K).

The critical nucleus radius r* (m) satisfies Equation (11):

(11) $$r^{*}=\frac{2\gamma }{\Delta G_{v}}$$

As indicated by Equations (6), (9) and (10) [33], the nucleation rate exhibits strong dependence on the critical nucleation energy barrier $\left(\Delta G^{*}\right)$ and the degree of undercooling. The equivalent undercooling ($\Delta T_{\mathrm{eff}}$) arises contributions from multiple factors, including thermal undercooling and constitutional undercooling [34]. Furthermore, increased cooling rate suppress solute diffusion, thereby enhancing the constitutional undercooling. In this study, the high cooling rate condition (150 K/s) employed during rheo-die casting significantly elevated the $\Delta T_{\mathrm{eff}}$. This strengthened the driving force for nucleation, yielding in a higher nucleation rate. Concurrently, the critical nucleus radius (r*) decreased, improving the thermodynamic stability of nuclei. Consequently, a larger population of newly formed small-sized nuclei was retained. This mechanism accounts for the abundance of fine secondary α_2_-Al phases observed in the microstructure of specimens presented in Figure 6a–c. Conversely, under the moderate cooling rate condition (15 K/s), the concentration field had relatively more time for diffusion, leading to reduced constitutional undercooling. This resulted in diminished driving force for growth. Simultaneously, an increase in r* was observed, indicating both lower nucleation rates for secondary α_2_-Al and poorer stability of nascent nuclei. Consequently, some secondary α_2_-Al phases formed at lower solid fractions underwent dissolution as the solid fraction increased, giving rise to the phenomenon of remelting, as illustrated in Figure 10.

Figure 10 further demonstrates contrasting growth behavior between primary α_1_-Al and secondary α_2_-Al phases under different cooling rates. Under high cooling rates, significant coarsening of secondary α_2_-Al phases was observed during solidification, while primary α_1_-Al phases sizes remained largely invariant. Conversely, under medium cooling rates, secondary α_2_-Al phases exhibited minimal growth or remelting, whereas primary α_2_-Al phases underwent marked coarsening. This divergence is explained by interface kinetics theory. For established nuclei, the solid-liquid interface velocity *v* (m/s) follows the Equation (12)

(12) v=M⋅ΔG

where *M* [m^4^/(J·s)] denotes interface mobility and ΔG (J/m^3^) represents the thermodynamic driving force governed by local temperature and composition. Equation (12) can be regarded as the sharp-interface version of Equation (1), with both sharing the same physical meaning, but the former is more convenient for our discussion here. At high cooling rates, solute trapping restricts chemical diffusion. Within infinitesimal time intervals (Δ*t*), temperature variations are negligible, and interfacial compositions remain effectively constant. Consequently, *v* approaches a time-invariant value. Under this regime, both primary α_1_-Al and secondary α_2_-Al phases grow radially at identical velocity *v*, yielding size increments of *2v·*Δ*t* per time step. Thus, small secondary phases achieve substantial relative growth (potentially multi-fold increases), whereas large primary phases exhibit limited absolute size changes due to their initial size. Under low cooling rates, prolonged solute redistribution elevates liquid supersaturation, suppressing secondary α_2_-Al nucleation and growth. Rare nucleation events possess insufficient ΔG to overcome interfacial energy barriers (Equation (10)), preventing sustained growth and triggering remelting. Consequently, solid fraction evolution becomes dominated exclusively by coarsening of pre-existing primary α_1_-Al phases. Despite reduced growth kinetics, significant coarsening occurs over extended solidification durations.

**Figure 11:**
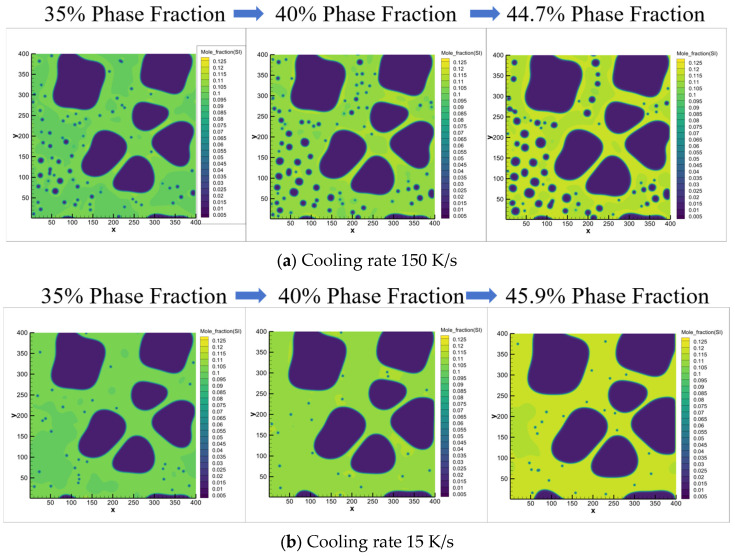
Solute field evolution dynamics of secondary α_2_-Al phase via phase-field simulation under different cooling rates: (**a**) 150 K/s, (**b**) 15 K/s.

To further validate the correlation between secondary α_2_-Al formation and process parameters (solid fraction/cooling rate) in rheo-die casting, solute field evolution was analyzed. Figure 11 presents simulated solute distributions during semi-solid processing, demonstrating how varying solid fractions influence secondary α_2_-Al formation. Notably, increasing cooling rates produced significantly divergent solute profiles at identical solid fractions. During rheo-diecasting solidification, high cooling rates induced pronounced solute trapping in the Al-7Si alloy [35]. This resulted in an effective partition coefficient (k_eff_) > the equilibrium coefficient (k_eq_), driving the actual liquid composition farther from equilibrium concentrations. Consequently, liquid supersaturation increased, amplifying constitutional undercooling and elevating total undercooling. These synergistic effects substantially promoted explosive nucleation of secondary α_2_-Al. Conversely, under medium cooling rates, reduced nucleation rates allowed sufficient time for solute diffusion [36] and redistribution during secondary α_2_-Al growth. This caused continuous enrichment of solutes in the liquid surrounding newly formed secondary α_2_-Al phases, thereby suppressing their subsequent growth.

**Figure 12:**
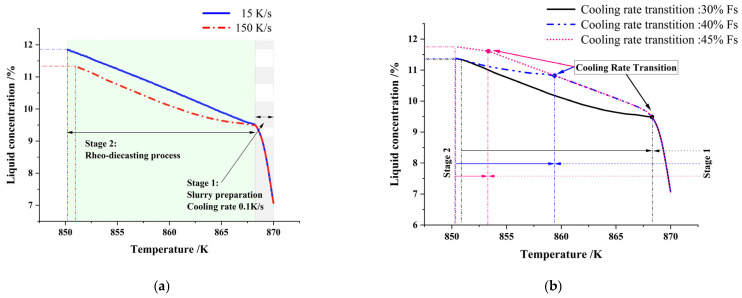
Phase-field simulated concentration-temperature evolution of residual liquid governed by slurry solid fractions and die-casting cooling rates: (**a**) Constant slurry fraction (30%, Stage Ⅰ 0.1 K/s): Profiles under high-cooling (150 K/s) vs. medium-cooling (15 K/s); (**b**) Constant die-casting rate (150 K/s): Profiles with differential initial solid fractions.

To elucidate the relationship between the growth kinetics of secondary α_2_-Al and liquid concentration, we statistically calculated the average liquid concentration at different temperatures based on the concentration distribution obtained from phase-field simulations. Figure 12a presents the average liquid concentration profile during Stage I solidification at a solid fraction of 30% under different cooling rates. Notably, at 150 K/s, the average liquid concentration remained significantly lower throughout the solidification process. Upon complete primary-phase solidification, residual liquid concentrations reached 11.38% (150 K/s) versus 11.82% (15 K/s), indicating a greater deviation from the eutectic equilibrium concentration under rapid cooling. Figure 12b demonstrates the effect of solid fraction (Stage I) on liquid concentration evolution at a fixed Stage II cooling rate of 150 K/s. As the Stage I solid fraction increased (30%, 40%, 45%), the average Si concentration in the remaining liquid exhibited a progressive elevation from 11.34% to 11.38% and 11.76%. Semi-solid slurries with lower solid fractions exhibited reduced average liquid concentrations compared to those with higher solid fractions. According to phase diagram analysis, the temperature differentials to the eutectic point were 22 °C (F_S_ = 30%), 13 °C (F_S_ = 40%), and 7 °C (F_S_ = 45%). Under identical cooling rates, secondary α_2_-Al formation persisted over longer durations in specimens with lower solid fractions. Conversely, higher initial solid fractions yielded elevated residual liquid concentrations during rheo-die casting, driving the Al-7Si system closer to the eutectic point. This consequently narrowed the solidification range, reduced undercooling, and suppressed explosive nucleation, ultimately decreasing both the population and dimensions of secondary α_2_-Al. The simulation outcomes show excellent correspondence with the experimental observations presented in Figure 6.

## 4. Conclusions

To elucidate the morphological evolution of α-Al phases during rheo-die casting solidification, considering the synergistic effects of cooling rate and solid fraction, an integrated experimental and phase-field methodology was employed. Principal outcomes and conclusions are summarized as follows:

(1) Based on two-stage cooling rate transition characteristics, a phase-field model coupling continuous cooling with explicit nucleation and the CALPHAD methodology was developed, enabling the dynamic simulation of continuous solidification microstructure evolution under cooling-rate variations. Integrated SEED slurry preparation and graded-cooling mold experiments were employed to establish a variable-cooling-rate and variable-solid-fraction condition. This approach enabled the quantitative analysis of α-Al morphological evolution during controlled rheo-die casting solidification.

(2) The evolution mechanism of primary α_1_-Al is predominantly governed by the solid fraction during Stage I slurry preparation. As the solid fraction increases from 30% to 45%, the average grain size increases from 70.4 μm to 111.1 μm. This coarsening results from Ostwald ripening under sufficient solute diffusion, where adjacent grains coalesce into larger rosette-shaped crystals, reducing the grain count. Kinetic analysis reveals that at 150 K/s, dimensional changes are limited by solute trapping and constrained growth time, while at 15 K/s, prolonged solidification with uniform solute redistribution enables sustained growth of primary α_1_-Al.

(3) The formation and morphology of secondary α_2_-Al during rheo-die casting are synergistically controlled by the cooling rate and solid fraction. At 150 K/s, secondary α_2_-Al is extensively nucleated as finely dispersed secondary phases surrounding primary phases. When the solid fraction is increased from 30% to 45%, its volume fraction is reduced from 4.78% to 0.33% while the phase size is decreased. Concurrently, the average Si concentration in the residual liquid rises from 11.34% to 11.76%. Solute field simulations reveal heterogeneous concentration distributions around α_1_-Al at high cooling rates. At 15 K/s, secondary α_2_-Al formation is observed minimally at 30% solid fraction and effectively suppressed at ≥40%, as nucleation is inhibited by sufficient solute diffusion throughout solidification.

(4) The nucleation and growth mechanisms of secondary α_2_-Al are explained through three aspects: nucleation driving force, solute trapping effect, and solid fraction. Regarding nucleation driving force, high cooling rates promote explosive nucleation by amplifying undercooling. Solute trapping under rapid cooling suppresses solute diffusion, intensifying compositional fluctuations and elevating effective undercooling—collectively enhancing nucleation. Conversely, an increased solid fraction elevates the solute concentration in the residual liquid, narrowing the solidification range and reducing undercooling, thereby suppressing secondary α_2_-Al nucleation.

## Figures and Tables

**Table 1:** Chemical composition of Al-7Si alloy used in this study (wt.%).

Alloy	Si	Fe	Al
Al-7Si	7.06	0.115	Bal.

**Table 2:** Thermodynamic and kinetic parameters of Al-7Si alloy.

Definition	Symbol	Value [Unit]
Phase-field mobility	*M_Φ_*	0.34 [m^3^·J^−1^s^−1^]
Anisotropy strength	ε	0.01
Initial solute concentration	*c_0_*	7 [mass%]
Diffusion coefficient for Si in liquid	*D_L_*	1 × 10^−9^ [m^2^·s^−1^]
Diffusion coefficient for Si in solid	*D_S_*	1 × 10^−13^ [m^2^·s^−1^]
Coupling constant	*λ*	5.0
Capillary length	*d*	0.2 [μm]
Solute partition coefficient	*k*	CALPHAD calculated
Liquidus	*T_Liquidus_*	CALPHAD calculated
Eutectic temperature	*T_eutectic_*	CALPHAD calculated
Melting point of pure solvent	*T_M_*	CALPHAD calculated
Nucleation parameter	*k_1_*	5 × 10^−2^
Nucleation parameter	*k_2_*	240

## Data Availability

The original contributions presented in this study are included in the article. Further inquiries can be directed to the corresponding author(s).
